# Factors associated with depression among people with cancer: Systematic umbrella review

**DOI:** 10.1017/S1478951526102247

**Published:** 2026-05-15

**Authors:** Barbara Zaccagnino, Arrigo Bettella, Anna Francesca Olivetti, Vittoria Cavalieri D'Oro, Marco Cruciata, Alfonso Tarricone, Claudia Iorio, Federica Sancassiani, Goce Kalcev, Michela Atzeni, Veronica Vacca, Alessandra Perra, Mariarita Monni, Angela Muscettola, Maria Giulia Nanni, Rosangela Caruso, Luigi Grassi, Martino Belvederi Murri

**Affiliations:** 1 Department of Neuroscience and Rehabilitation, University of Ferrara, Ferrara, Italy; 2 Hospital University Psychiatry Unit, S. Anna Hospital/Local Health Trust, Ferrara, Italy; 3 University of Ferrara School of Medicine, Ferrara, Italy; 4 Department of Medical Sciences and Public Health, University of Cagliari, Cagliari, Italy; 5 Center of Liaison Psychiatry and Psychosomatics, University Hospital of Cagliari, Cagliari, Italy

**Keywords:** Cancer, depression, risk factor, protective, risk prediction, social factors

## Abstract

**Introduction:**

Depression in cancer patients is a common condition that poses significant challenges for prognosis, treatment adherence, and quality of life. Its onset reflects the interplay of diverse biological, psychological, and social factors, which has been the focus of numerous studies.

**Methods:**

We identified both systematic and non-systematic reviews examining cross-sectional or prospective studies reporting associations between DAFs and depression. We extracted data relative to DAFs, as well as the direction and statistical significance of the reported association. Consistency of findings was assessed by estimating the proportion of concordant studies (PCS) for each DAF. Methodological quality and risk of bias were assessed using a standardized tool.

**Results:**

We identified 73 reviews (26 systematic and/or meta-analyses, 47 narrative) encompassing 514 unique primary studies, reporting the associations between depression and 198 distinct DAFs. DAFs were grouped into six domains (sociodemographic, cancer-related, somatic, psychological, biological-genetic, and other). The strongest associations (PCS ≥ 75% and ≥5 studies) were observed for sociodemographic factors (e.g., high social support, being unmarried), inflammatory markers (IL-6, TNF-α, CRP), psychological factors (e.g., history of depression, distress, anxiety), and somatic factors (e.g., fatigue, low functional status, malnutrition). When restricting analyses to prospective studies, consistent associations emerged for cancer-related physical symptoms and time dedicated to patient communication.

**Conclusions:**

Depression in cancer is multifactorial, with physical and psychosocial factors likely iteracting dynamically. Prospective studies are still greatly needed. Further research on risk and protective factors may facilitate risk stratification, early diagnosis and patient management.

## Introduction

Depression is highly prevalent and debilitating among individuals affected by cancer, yet its etiology is multifactorial, and the timely identification of depressive syndromes remains challenging (Caruso et al. 2017b; Belvederi Murri et al. 2022).

Major depression adversely affects both the quality of life of cancer patients and cancer prognosis (Bai 2022; Caruso et al. 2017a). The onset of depression is influenced by a wide range of factors, which are increasingly being elucitated, commonly grouped into biological, psychological, and social domains. For instance, among biological factors, increased secretion of tumor-related inflammatory cytokines (Patton et al. 2021), as well as the effects of cancer treatments – including corticosteroids, exogenous cytokines, hormonal therapies, and chemotherapeutic agents – have been associated with increased depressive symptoms (Korsten et al. 2019; Wen et al. 2019; Ibrahim et al. 2021). Physical pain also represenys both a biological and psychological risk factor for depressive syndromes (Wen et al. 2019; Lee et al. 2023). With regard to psychological factors, a cancer diagnosis constitues a profoundly distressing life event and coping abilities vary across individuals depending on socio-cultural and personal variables. Levels of hope and resilience may function as either protective or risk factors for depression (Smith et al. 2018). Depression in cancer patients can also reshape individual’s perception of their social role, highlighting the importrance of social determinants in the development of depression (Lee et al. 2023). Family environment and social support exert a significant influence, either exacerbating or mitigating depressive symptoms (Riedl and Schüßler 2022).

Building sound causal hypotheses or risk-prediction tools begins with identifying all variables that show any statistical link with depressive outcomes, whether detrimental or protective (Cattelani et al. 2019; Belvederi Murri et al. 2022). However, the conventional terms “risk factors” and “protective factors” often imply a degree of causal certainty that observational research rarely supports (Huitfeldt 2016; Schooling and Jones 2018). To address this, we adopt an unambiguous nomenclature: variables examined in cross-sectional analyses are referred to as correlates; those tested in prospective designs as predictors; and the inclusive term associated factors is used for both.

The aim of this umbrella review was to synthesize secondary literature, incluiding narrative reviews, systematic reviews and meta-analyses, on factors associated with depression (depression-associated factors, DAFs) among adults with cancer, and to quantify the consistency of each association.

### Objectives and Significance of the Results

This systematic umbrella review examines the secondary literature on depression associated factors (DAFs) in adults with cancer and evaluates the consitency of their association.

## Methods

We summarised findings from multiple research syntheses by conducting an umbrella review, a methodology that systematically examines existing reviews and meta-analyses to compare and contrast findings, and provide a comprehensive overview (Fusar-Poli and Radua 2018). We followed the JBI Manual for Evidence Synthesis (Aromataris et al. 2024).

### Search strategy and information sources

This umbrella review followed PRISMA guidelines (Page et al. 2021). We systematically searched PubMed for secondary literature published between 1 January 1994 and 31 December 2024, a 30-year time frame corresponding to the introduction of DSM-IV diagnostic criteria and intended to reduce methodological heterogeneity.

Studies on DAFs in adult cancer patients were identified using the following search string, with appropriate filters: (depression AND cancer) AND (review OR meta-analysis). We included only studies published in English and involving adult populations (≥18 years). The search was not further restricted by including terms such as “risk AND/OR protective factors.” Additionally, the reference lists of included studies were manually screened, and supplementary searches were conducted on Google Scholar, examining the first 10 pages of results to identify potentially relevant studies not indexed in PubMed. No additional studies were identified.

### Eligibility criteria

Studies were included if they: (1) were systematic, non-systematic reviews or meta-analyses; (2) examined associations between biological, psychological, or social DAFs (exposure) and depression (outcome) in adults with cancer; (3) were published in English. Inclusion was not restricted by design of the primary studies (e.g., cohort, case-control, cross-sectional), recruitment setting (e.g., primary care, hospital), participant characteristics, cancer type (solid or hematologic), disease stage, or the definition, assessment or severity of depression.

### Selection process

After removing duplicates, titles and abstracts were screened independently by two reviewers for eligibility and were either retained for full-text assessment or excluded. In the second phase, the full text of potentially relevant articles was assessed for inclusion. Disagreements between reviewers (AB, BZ) were resolved through consultation with a third reviewer (MBM).

### Data extraction and synthesis methods

Data extraction proceeded in two sequential stages. First, we extracted data from systematic reviews and meta-analyses, defined as studies reporting a search strategy and eligibility criteria. In this stage, we used a piloted Excel in which information was recorded for each eligible review, with one DAF per row. To prevent double-counting, each primary study was entered once only in a master log structured with one row per study. Two researchers independently extracted data on DAFs from each reviewFor each reviw, we collected: author, publication year, study design (systematic review vs. meta-analysis), number of included studies (total and longitudinal), participant eligibility criteria, study setting, cancer type and stage, details of methodological appraisal of primary studies; the number of of studies related as high quality , and the operational definitions used for each DAF.

The second stage focused on factor-level data. For each DAF listed in the descriptive tables of the source reviews, the same two reviewers independently extracted the number of primary studies reporting a positive, inverse or non-significant association with depression, using the significance threshold adopted by the originating review (typically *p* < 0.05 in the fully adjusted model). We also recorded the number of studies that examined the association prospectively. When the source review included a meta-analysis, the extraction captured the details of the statistical model applied (fixed, random or hierarchical), the pooled effect size and corresponding 95% confidence (CI) or credible intervals (CrI). These factor-level data were entered into a second spreadsheet, structured with one row per DAF. Disagreements at either extraction stage were resolved by discussion; a third reviewer (MBM) adjudicated if consensus could not be reached..

Following data extraction, two reviewers (BZ and AFO) consolidated variables that represented the same underlying construct but differed in wording, measurement scale or anatomical focus. For instance, pain reported across different body sites (e.g., bone, abdominal, procedure-related) and pain interference scores were merged into a single DAF labeled “pain.” This grouping was performed iteratively, with each reviewer working independently and subsequently reconciling discrepancies through discussion; a third reviewer (MBM) adjudicated unresolved cases. In addition, the direction of each association was standardized. When complementary categories of the same factor yielded mirror-image findings – for example, one study reporting that older age predicted higher depression scores and another showing that younger age predicted lower scores – the latter result was recoded so that both observations contributed to evidence that older age was positively associated with depression. This sign-reversal procedure ensured that every DAF retained a single, consistent direction of effect across the aggregated dataset, thereby facilitating meaningful comparison of the consistency and strength of evidence among factors.

During the analysis, several binary factor pairs (e.g. “female gender” vs. “male gender”) were identified as being assessed within the same studies. To minimize data fragmentation and ensure a clear, unambiguous interprettaion, one factor from each pair (e.g., “female gender”) was selected as the reference category. The number of studies identifying it as a risk factor was then calculated and the number of studies identifying the comolementary factor (e.g., “male gender”) as a risk factor was subtracted. This yielded a net value reflecting the contrast between opposing factors, providing a single, meaningful unidirectional indicator of association.

To synthesize results, we calculated the proportion of concordant studies (PCS), defined as the number of primary studies reporting a *statistically significant* association in the same (sign-standardized) direction divided by the total number of studies examining that factor (Fernandez et al. 2025). Given the expected small number of studies per DAF formal meta-analysis or the application of more recent criteria was not considred (Gosling et al. 2023).

For narrative reviews, we limited data extraction to author, publication year, cancer type, and names of DAFs.

### Study risk of bias assessment

To help identify and mitigate reporting bias, this umbrella review followed a pre-established protocol outlining the research question and methodology. Methodological quality and risk of bias of this Umbrella Review were assessed using the Joanna Briggs Institute (JBI) Critical Appraisal Checklist for Systematic Reviews and Research Syntheses (Hilton 2024) (Table S2). The checklist includes 11 items answered as “yes,” “no,” “unclear,” and “not applicable (NA). We classified total scores ≤ 4 as low quality, 5-7 as moderate quality, and scores over 8 as high quality. Two reviewers (BZ, AFO) independently conducted the appraisal, with disagreements resolved by a third reviewer (MBM).

### Reporting bias assessment

Potential sources of bias were assessed using the JBI Critical Appraisal Checklist (Hilton 2024), with attention to selection bias, publication bias, variability in quality appraisal methods, and redundancy across primary studies.

## Results

### Study selection

**Figure 1.**
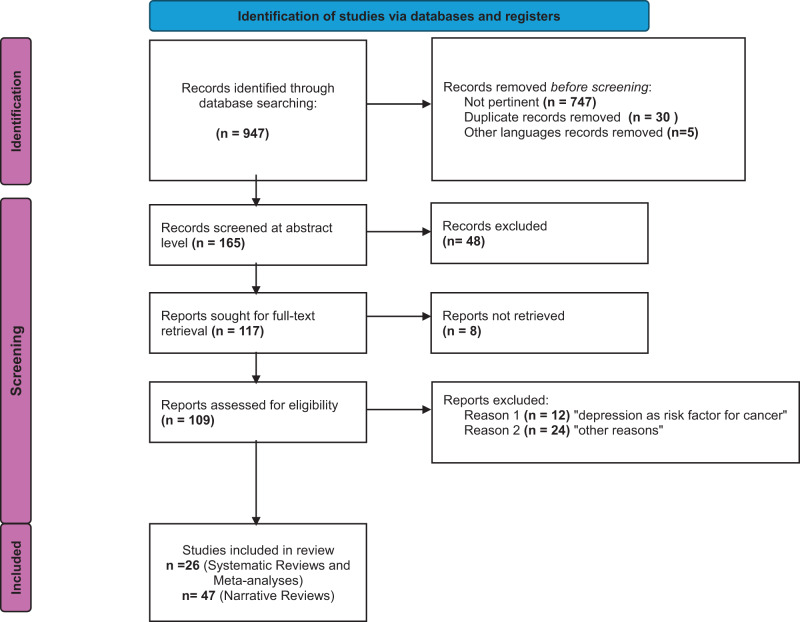
Prisma flow diagram.

Figure 1 shows the PRISMA flow-chart. The search identified 165 potentially relevant records. After removal of duplicates, the remaining 132 records were screened. Following the exclusion of 23 records , the full texts of 109 articles were assessed for eligibility. Of these 36 studies were excluded at the full-text stage; reasons for exclusion are reported in Table S1. A total of 73 reviews were included, including 26 systematic reviews and/or meta-analyses) (Laird et al. 2009; Bellardita et al. 2015; Suppli et al. 2015; Nead et al. 2017; Pop et al. 2017; Smith et al. 2018; Zhang et al. 2018; Freitas and Campos 2019; Korsten et al. 2019; Sforzini et al. 2019; Wen et al. 2019; Siebert et al. 2020; Ayubi et al. 2021; Batra et al. 2021; Ibrahim et al. 2021; Padmalatha et al. 2021; Patton et al. 2021; Alwhaibi et al. 2022; Chair et al. 2022; McFarland et al. 2022; Riedl and Schüßler 2022; Alexander et al. 2023; Beck et al. 2023; Kitashita and Suzuki 2023; Lee et al. 2023; Szabados et al. 2023) and 47 narrative reviews.

The diagram illustrates the systematic process used to identify, screen, and include studies for the umbrella review.

### Study characteristics

We included 73 studies (Table 1 and S3): 18 systematic reviews without meta-analysis, 6 systematic reviews with meta-analyses, and 2 systematic meta-analyses embedded within other study types. After deduplication, these reviews comprised 514 unique primary studies, with 49 duplicates identified from an initial pool of 563, corresponding to a duplication rate of 8.7%. For 29 primary studies, information on examined DAFs was unavailable, resulting in a final sample of 485 primary studies for analysis. In total, we identified 964 DAFs, corresponding to 290 unique DAFs (initially distinguishing treatment types, surgeries, and tumor locations). After regrouping and consolidating similar factors (e.g., age groups, gender, personality traits), the final set included 198 unique DAFs. The most frequently analyzed risk factors are illustrated in Figure S1. Among the 26 systematic reviews and meta-analyses, considerable heterogeneity was observed in cancer types: 1 thyroid, 1 head and neck, 1 intracranial tumor, 1 testicular, 2 pancreatic, 5 prostate, 3 breast, 1 gastric, 1 colorectal, and 10 non-specific cancers. Additionally, 4 studies focused on long-term survivors, 2 on metastatic and advanced-stage tumors, 2 on non-metastatic tumors, and 18 did not specify tumor stage as an inclusion criterion. DAFs were classified into six domains: sociodemographic, cancer-related, somatic, psychological, biological-genetic, and other.

**Table 1.** Systematic reviews and meta-analyses characteristics

Citation	Study design, n primary studies, n longitudinal	Study settings, participant eligibility	Associated factors names	Methodological appraisal, n high-quality studies
Alexander et al. (2023)	Systematic review, 68, 25	Mixed, only long-term survivors, thyroid cancer	Older age, female, being unmarried, living alone, low education status, Hispanic ethnicity, Asian ethnicity, lower income, being unemployed, out of pocket payment for treatment, high TNM stage, cancer related physical symptoms, current active disease or cancer recurrence, longer time since diagnosis, later/first year postoperative/increased time since surgery survivorship stage, hypothyroidism/hypoparathyroidism treatment complications, levothyroxine withdrawal treatment complication, high TSH treatment complication, radioactive iodine treatment, thyroidectomy treatment, hemithyroidectomy treatment, active surveillance treatment, lobectomy treatment, physical comorbidities, fear of cancer recurrence, fear of future treatment options, negative diagnosis treatment outlook, dedicated information time, receiving psychological and behavioral nursing intervention	NR, NR
Alwhaibi et al. (2022)	Systematic review, 33, 19	Mixed, prostate cancer, ADT treatment	ADT therapy	NR, NR
Ayubi et al. (2021)	Systematic review, 34, 1	Primary care, cancer patients with PICCs, mainly stages 3 and 4	COVID-19 pandemic	NR, NR
Batra et al. (2020)	Systematic review, 14, 13	Specific cancer, prostate cancer, only with metastases	ARAT vs. prednisone therapy, ARAT vs. placebo therapy, ARAT vs. bicalutamide therapy	GRADE, 0
Beck et al. (2023)	Systematic review, 14, 3	Mixed, age > 18 y	Rumination	Effective Public Health Practice Project (EPHPP), 4
Bellardita et al. (2015)	Systematic review, 4, 1	Mixed, prostate cancer, no criteria based on stage	Active Surveillance for Prostate Cancer	NR, NR
Chair et al. (2022)	Systematic review, 22, 1	Mixed, cancer treatment, no criteria based on stage	Genetic marker-511 C > T SNP (IL-1β) presence in breast cancer, genetic marker rs1800795 (IL-6) presence in breast cancer, genetic marker rs2069840 (IL-6) presence in breast cancer, genetic marker rs1799964 (TNF-α) presence in breast cancer, genetic marker rs9376268 (IFNGR1) in breast cancer, genetic marker 5-HTTLPR deletion in breast cancer, genetic marker rs6265 (BDNF) presence in breast cancer, genetic marker rs9296158 (FKBP5) presence in gastric cancer, genetic marker rs9470080 (FKBP5) presence in gastric cancer, genetic marker rs10781582 (BNIP3) presence in gastric cancer, genetic marker 251 T > A SNP (IL-8) presence in lung cancer, genetic marker GSTM1 deletion, genetic marker 5-HTTLPR deletion, genetic marker BClI SNP (NR3C1) presence, genetic marker rs25531 SNP of 5-HTTLPR deletion in colorectal cancer	The STrengthening the REporting of Genetic Association (STREGA) checklist, NR
Freitas and Campos (2019)	Systematic review, 2, 0	Mixed, gastric cancer, no criteria based on stage	PUFAs food supplements	NR, NR
Kitashita and Suzuki (2023)	Systematic review, 13, NR	Mixed, chemotherapy, molecular-targeted therapy, endocrine therapy, immunotherapy treatment, no criteria based on stage	High levels of hope	The Joanna Briggs Institute (JBI) checklist, NR
Korsten et al. (2019)	Systematic review, 35, 35	Mixed, head and neck cancer, age > 18 y, no criteria based on stage	Female, older age, being married, living alone, being unemployed, higher income, high educational level, current smoker, alcohol, higher disease stage, chemotherapy, radiotherapy, pharmacological treatment, low performance (Karnofsky), dry mouth, sense problems, poor sexuality functioning, pain killers assumption, speech problems, tumor location, physical comorbidities, neck disability, satisfaction with looks, diet, symptoms of depression, anxiety disorder, sense of humor, childhood trauma, number of life events, avoidant coping, self-blame, social support, higher self-esteem, high locus of control, bad communication with medical team, cancer-related symptoms, illness perception, satisfaction with cancer information, pain	12-item quality assessment scoring list, 11
Ibrahim et al. (2021)	Systematic review and meta-analysis, 8, 8	Mixed, breast cancer without brain metastases, 18–69 y, taxane treatment	Taxane therapy	Newcastle-Ottawa Scale, 5
Laird et al. (2009)	Systematic review, 14, NR	Mixed, no criteria based on stage	Female, older age, pain, pain intensity, pain duration	Scottish Intercollegiate Guidelines Network (SIGN) checklist, NR
Lee et al. (2023)	Systematic review, 31, 6	Mixed, age > 65 y, no criteria based on stage	Female, fatigue, pain, cancer-related symptoms, malnutrition, longer time since diagnosis, ADT, chemotherapy, radiotherapy, surgery, advanced cancer, physical comorbidities, low physical functioning/impaired ADLs, anxiety, pre-cancer emotional frailty, depressive symptoms at baseline, being unmarried, living with children, lower income, older age, high educational level, optimism, high social support, spirituality, being inpatient, smoking, alcohol, physical activity	The Joanna Briggs Institute (JBI) checklist, 15
McFarland et al. (2022)	Systematic review and meta-analysis, 73, 23	Mixed, no criteria based on stage	Increased inflammatory markers before curative-intent surgery, increased inflammatory markers after curative-intent surgery, increased inflammatory markers in patients with advanced cancer receiving palliative treatment, increased inflammatory markers in patients who were receiving chemotherapy, increased inflammatory markers in patients who were receiving radiotherapy, increased inflammatory markers in survivorship setting, IL-6, IL-6 (localized cancer), IL-6 (metastatic cancer), IL-6 and depressive symptoms in studies with depression as the primary endpoint, IL-6 and depressive symptoms in studies with depression as a non-primary endpoint, TNF, TNF (localized cancer), TNF (metastatic cancer), TNF and depressive symptoms in studies with depression as the primary endpoint (16 studies)	Modified Downs and Black Checklist adapted for observational studies, NR
Nead et al. (2017)	Systematic review and meta-analysis, 26, 13	Mixed, prostate cancer, ADT treatment, no criteria based on stage	ADT therapy	Newcastle-Ottawa Scale, NR
Padmalatha et al. (2021)	Systematic review and meta-analysis, 9, 9	Mixed, breast cancer, total mastectomy vs. breast reconstruction, no criteria based on stage	Total mastectomy with no BR	The Joanna Briggs Institute (JBI) checklist, 9
Patton et al. (2021)	Systematic review, 9, 3	Mixed, age > 18 y, only incurable cancer (metastatic cancer or locally advanced cancer treated with palliative intent)	High IL-6 level, IL-8 level	Downs and Black Checklist (MDBC), 6
Pop et al. (2017)	Systematic review, 3, NR	Mixed, pancreatic cancer, no criteria based on stage	IL-6	NR, NR
Riedl and Schüßler (2022)	Systematic review, 40, 24	Mixed, age > 18 y, no criteria based on stage	Older age, female, Caucasian ethnicity, African/American ethnicity, low educational level, living alone, lower income, lower social support, cancer-related symptoms, physical comorbidities, low physical functioning, pain, adjuvant therapy, chemotherapy, definitive therapy, watch and wait, radiotherapy, cancer type, high TMN or metastases, previous depression, psychiatric history, anxiety, hopelessness, introverted personality, maladaptive coping, unhealthy behavior, low emotional functioning, stressful life events, worries, neuroticism, rumination, higher spirituality, agreeableness, self-esteem, optimism	Downs e Black Checklist (MDBC), 27
Sforzini et al. (2019)	Systematic review, 10, NR	Pancreatic adenocarcinoma, melanoma, renal cell carcinoma, breast cancer, colon adenocarcinoma, brain tumor, no criteria based on stage	Kynurenine pathway alterations, increased level of kynurenine, altered tryptophan metabolism, increased K/T ratio, increased level of IDO or TDO, increased level of neopterin	NR, NR
Siebert et al. (2020)	Meta-analysis, 6, 1	Mixed, prostate cancer, ADT treatment, no criteria based on stage	ADT therapy	The Joanna Briggs Institute (JBI) checklist, 9
Smith et al. (2018)	Systematic review, 21, NR	Mixed, age > 18 y, testicular cancer, only long-term survivors	Being unmarried, low social support, fatigue, negative health behaviors, avoidant coping, hopeless coping, poorer sexual functioning, previous psychological distress, altered body image and sense of masculinity, older age, low educational level, hormonal level alteration, longer time since diagnosis, treatment type, comorbidities, smoking, low physical activity, anxiety, neuroticism, self-reported cognitive problems	14-item checklist designed to assess quality of healthcare intervention studies, 10
Suppli et al. (2015)	Meta-analysis, 6, NR	Mixed, colorectal cancer, after cancer	Serotonin transporter gene (SS genotype and SL genotype vrs LL genotype)	NR, NR
Szabados et al. (2023)	Systematic review and meta-analysis, 9, NR	Mixed, intracranial cancer, only long-term survivors	Supratentorial (SUP) brain tumor, infratentorial (INF) brain tumor	Quality in Prognostic Studies 2 (QUIPS − 2), risk of bias assessment), NR
Wen et al. (2019)	Systematic review, 43,17	Mixed, age > 18y, chemotherapy, no criteria based on stage	Older age, low educational level, female, nuclear family, being unemployed, lower income, history of abortion, living alone, hispanic ethnicity, family history of cancer, pain, inadequate nutritional intake, low BMI score, fatigue, sleep disturbance, appetite loss, no weight change, frequency of sexual activity, menopausal symptom, low sexual function, concentration of CA125, TRACP5A, WBC, number of monocyte, restless leg syndrome, gastrointestinal symptoms, numbness, social support, anxiety, distress, perceived stress, negative beliefs, self-efficacy, high physical functioning, fighting spirit, high hope level, cancer-related symptoms severity, cancer stage, cancer type, time since beginning of chemotherapy, no response to chemotherapy, chemotherapy-induced alopecia	Agency for Healthcare Research and Quality (AHRQ) checklist, 17
Zhang et al. (2018)	Systematic review and meta-analysis, 16, 16	Mixed, breast cancer, TM vs. BCS vs .BR, no metastases	Therapy (TM vs. BR vs. BCS)	Newcastle-Ottawa Scale, 16

Abbreviations: NR, not reported; TNM, Tumor, Node, Metastasis; TSH, thyroid stimulating hormone; ADT, androgen deprivation therapy; ARAT, novel androgen receptor-axis-targeted; PUFA, polyunsaturated fatty acids; BR, brachytherapy; TNF, tumor necrosis factor; BMI, body mass tndex; TM: total mastectomy; BR, breast reconstruction; BCS, breast conserving therapy.

The figure illustrates the association between sociodemographic factors and depression in the oncology setting, showing the direction of each association (risk vs. protective), the number of primary studies, and the percentage of concordant. associations.

### Factors associated with depression in patients with cancer

Figure S1 shows the 10 most frequently investigated risk factors in primary studies.

#### Sociodemographic factors

**Figure 2.**
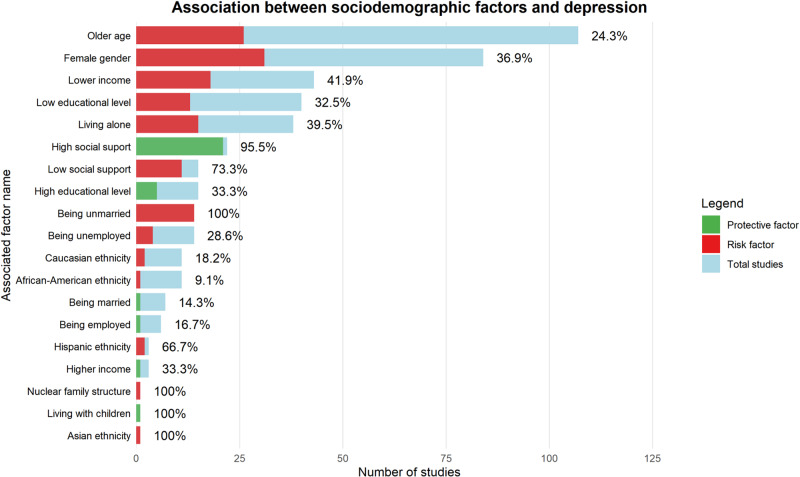
Association between sociodemographic factors and depression.

Among sociodemographic factors (Figure 2), age was examined in 107 primary studies, with most defining “older age” variably; only one review specified it as > 65 years. A significant association with depression was found in 24.3% of these studies, decreasing to 10.3% in longitudinal analyses. Gender was examined in 84 studies (all but two included females), with female gender associated with higher depression risk in 36.9% of studies (37.5% in longitudinal ones).

The figure illustrates the association between sociodemographic factors and depression in the oncology setting, showing the direction of each association (risk vs. protective), the number of primary studies, and the percentage of concordant associations.

Marital and family status were assessed across multiple dimensions: 14 studies reported a positive association between being unmarried and depression; among 7 longitudinal studies evaluating being married as a protective factor, only one confirmed a reduced risk. Living alone was examined in 38 studies, with approximately 40% reporting it as a risk factor, a findng consistent across longitudinal studies.

Educational level was assessed in 40 studies, of which 13 (32.5%), including one longitudinal study, reported an association between low educational attainment and depression. Among 15 studies evaluating higher education as protective, 33.3% (five studies) supported this association; however only 10% among longitudinal studies did so. Definitions varied, with only one study specifying “higher education” as completion of at least high school.

Employment status was examined in 14 studies on unemployment, of which 28.6% reported a positive association with depression. Among six longitudinal studies exploring employment as a protective factor, only one (16.7%) confirmed this association. Economic status was assessed in 43 studies, with 42% identiifying low income as a risk factor for depression (including one longitudinal), while 33.3% of longitudinal studies supported high income as a protective factor.

Ethnicity was examined in 25 studies: 11 on African Americans, 11 on Caucasians, 3 on Hispanics, and 1 on Asians. Positive associations with depression were found in 9.1%, 18.2%, 66.7%, and 100% of these studies, respectively.

Finally, social support was assessed in 15 studies examining low support, with 73.3% reporting a positive association with depression. Conversely, 95.5% of 22 studies (including 9 longitudinal studies) confirmed high social support as a protective factor.

#### Biological and genetic factors

Regarding inflammatory biomarkers, 36 studies assessed general indicators of inflammation across various cancer stages, with 72.2% reporting a positive association between increased inflammation and depressive symptoms. The risk was notably higher during chemotherapy, radiotherapy, and preoperative phases. Tumor necrosis factor alpha (TNF-α) was identified as a risk factor in 87.5% of studies (21/24), while C-reactive protein (CRP) showed a significant association in 76.6% (10/13), supported by a meta-analysis demonstrating a moderate effect size (SMD = 0.57), which increased to 0.75 in metastatic cases. Interleukin-6 (IL-6) was linked to depressive symptoms in 76.6% of studies (40/52). Interleukin-8 (IL-8) appeared potentially protective in 33.3% (1/3) of studies; a meta-analysis reported a moderate protective effect in the general cancer population (SMD = –0.59) and a large effect in metastatic patients (SMD = –0.90).

In the context of neuro-immune axis alterations, all 12 studies examining tryptophan metabolism dysregulation supported an association with depression. Assessed biomarkers included increased indoleamine 2,3-dioxygenase (IDO) activity (*n* = 1), elevated kynurenine/tryptophan (K/T) ratio (*n* = 2), increased kynurenine levels (*n* = 5), tryptophan metabolism alterations (*n* = 2), and elevated neopterin levels (*n* = 2). Additionally, 10 primary studies on the kynurenine pathway reported significant associations in 40% of cases.

Finally, one systematic review and one meta-analysis investigating genetic polymorphisms found no significant association with depression (Figure S2).

#### Factors of the “somatic” and “other” domains

The factor “fatigue” showed a significant association in 8 out of 9 studies, including 1 longitudinal study (88.9%).

Functional status was analyzed in 26 studies, of which 24 found a significant association (83.3%); 2 of the 24 primary studies identified high functional status as a protective factor.

Regarding to sexual functioning, 4 in 5 studies identified a significant association between poor sexual functioning and depressive symptoms, while 1 study reported that frequent sexual activity was associated with a reduced risk of depression.

Body image was examined in 3 studies assessing poor body image perception: one study focused on testicular cancer survivors, and two on patients with head and neck cancer.

Physical activity and unhealthy lifestyle were examined in 12 studies, 6 of which identified a significant association with a sedentary lifestyle. An additional study reported engagement in physical activity as a protective factor.

The factor “comorbidity” was significantly associated with depression in 31/40 primary studies (77.5%), including 3 longitudinal studies.

Pain was analyzed in 37 studies, with 24 reporting a significant association (65%). This proportion increased to 80% when considering pain intensity and chronicity, which were directly assoiated with greater severity of depressive symptoms.

Cancer-related symptoms were examined in 39 studies, including 5 longitudinal ones, with 22/39 (56.4%) reporting a significant association. Thirty studies assessed symptoms in general terms. One study focusing on head and neck cancers reported a 50% significance rate for symptoms such as xerostomia and sensory or communication difficulties. Additionally, two studies investigating Restless Legs Syndrome (Willis-Ekbom disease) reported a 100% association among patients undergoing chemotherapy.

Nutrition was examined in 11 primary studies. Eight studies (including 2 longitudinal ones) investigated malnutrition as a risk factor for depression, with all reporting significant associations. The remaining three studies identified protective effects of adherence to healthy dietary patterns, maintenance of body weight, and the use of Omega-3 polyunsaturated fatty acids.

Sleep disorders (insomnia, hypersomnia, and daytime sleepiness) were analyzed in five primary studies, with four (80%) reporting significant associations with depression.

**Figure 3.**
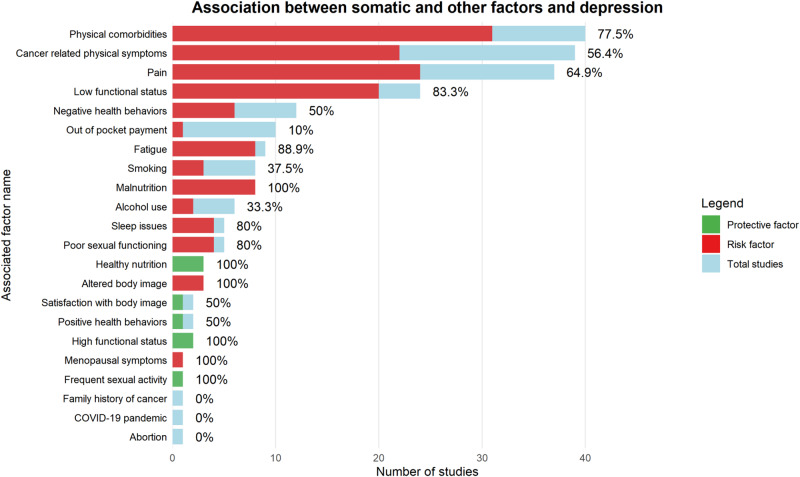
Association between somatic and other factors and depression.

Substance use was analyzed in 14 primary studies: tobacco use was associated with an increased risk of depressive symptoms in 3 of 8 studies (37.5%), while alcohol consumption was significantly associated with a higher risk of depression in 2 of 6 studies (Figure 3).

The figure illustrates the association between factors in the “somatic” and “other” domains and depression in the oncology setting, showing the direction of each association (risk vs. protective), the number of primary studies, and the percentage of concordant associations

#### Cancer-related factors

Regarding tumor site, 2 of 13 primary studies (15.4%) confirmed a significant association with increased risk of depression. In terms of cancer stage, 19 of 64 primary studies (29.7%), including 8 longitudinal studies, reported a significant association between advanced stage or metastatic disease and depression.

A total of 243 primary studies examined the role of cancer treatments in the development of depression. Among 33 studies on active surveillance (“watchful waiting”), one identified it as a risk factor, while six (19%) found a significant protective association, particularly in patients with thyroid or prostate cancer. Surgical treatment was evaluated in 113 studies: five described surgery – both radical and less invasive approaches – as protective., whereas, 84 studies considered it a potential risk factor, with 20 (23.8%) reporting a significant association. Of these, 18 studies (including two meta-analyses) focused specifically on total mastectomy (TM). Androgen Deprivation Therapy (ADT) was examined in 36 studies, with 25 (69.4%), including 10 longitudinal studies demonstrating a significant association with increased depressive symptoms. Regarding chemotherapy, 11 out of 25 studies reported a positive association with depression onset (44.0%, longitudinal: 36.8%). Radiotherapy was identified as a significant risk factor in 12 of 36 studies (33.3%)

A total of 51 primary studies investigated the role of time since diagnosis or treatment. Among these, 1 of 9 studies (11.1%) reported an increased risk of depression in the immediate postoperative period. Conversely, 10 out of 42 studies (23.8%) identified longer time since diagnosis or treatment as a protective factor against depressive symptoms.

Among the 49 primary studies investigating the impact of negative illness perceptions (e.g., fear of recurrence, concerns about treatment efficacy) on the development of depressive symptoms, 34.7% (17 studies, including 5 longitudinal ones) reported a significant association.

**Figure 4.**
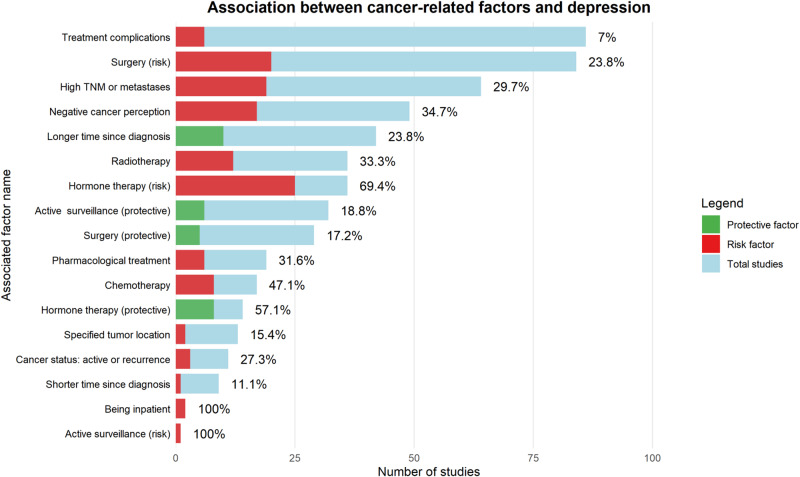
Association between cancer-related factors and depression.

Finally, 3 out of 11 studies (27.3%) identified cancer recurrence as a risk factor for depression (Figure 4).

The figure illustrates the association between cancer-related factors and depression in the oncology setting, showing the direction of each association (risk vs. protective), the number of primary studies, and the percentage of concordant associations.

**Figure 5.**
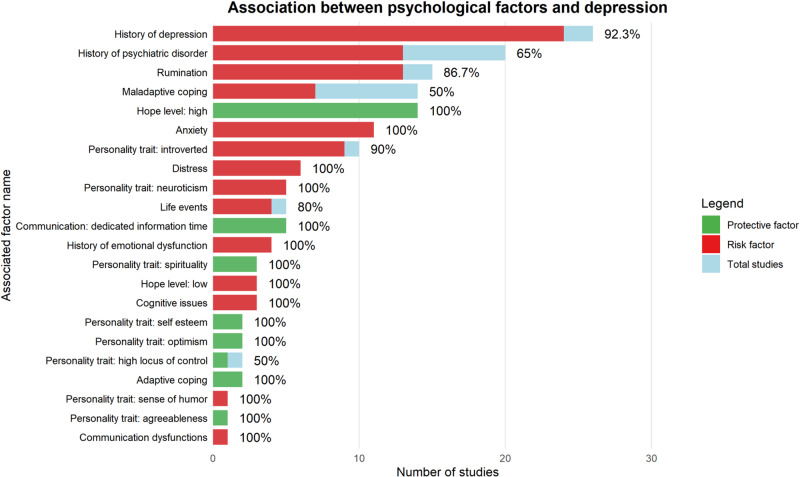
Association between psychological factors and depression.

The figure illustrates the association between psychological factors and depression in the oncology setting, showing the direction of each association (risk vs. protective), the number of primary studies, and the percentage of concordant associations.

#### Psychological factors

Coping styles were grouped as *adaptive* (e.g., fighting spirit, acceptance) and *maladaptive* (e.g., denial, avoidance). Adaptive coping was protective in 2/2 studies (100%), whereas maladaptive coping was associated with increased risk of depression risk in 7/14 studies (50%).

Hope was protective in all 14 studies (100%), while hopelessness predictive of depression in all three studies (3/3, 100%). One longitudinal study linked poor clinician–patient communication to depression, whereas 4 of 5 studies (80%) reported open and effective communication as protective.

Regarding personality traits, agreeableness and internal locus of control were protective (2 studies), while high self-esteem and optimism were associated with reduced risk in all four studies (4/4, 100%). Neuroticism was associated with depression in all 5 studies, and introversion in 9 of 10 (90%).

Spirituality was protective in all three studies (3/3, 100%). Among stress-related variables, 4 of 5 studies (80%, including 3 longitudinal) linked stressful life events to depression. Emotional dysregulation was identified as a risk factor in all four studies (4/4, 100%). A history of major depression predicted relapse in 24 studies (92.3%, longitudinal: 80%), and prior psychiatric disorders were associated with depression onset in 13of 20 studies (65%).

Distress (6/6, inccluding 1 longitudinal) and anxiety (11/11, inclduing 3 longitudinal) were consistently associated with increased depression risk. Rumination was linked to depression in 13 of 15 studies (86.7%), including all longitudinal ones (100%).

#### Non-systematic reviews

**Figure 6.**
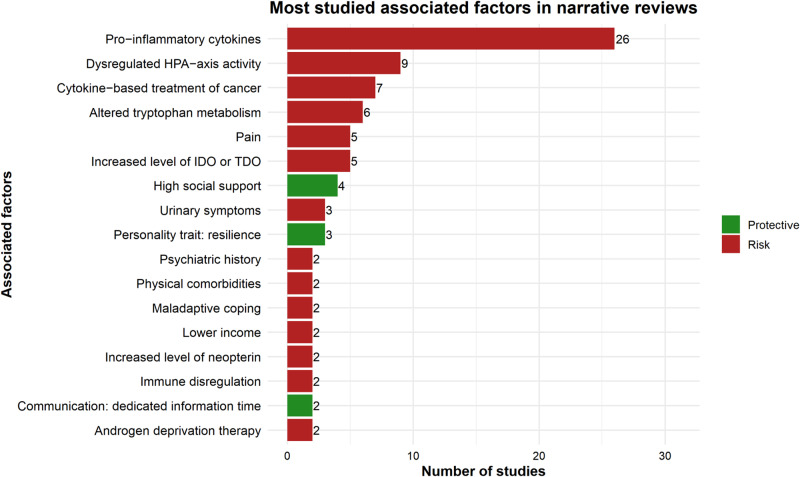
Most studied associated Factors in Narrative Reviews.

Figure 6 summarizes the most frequently reported risk and protective factors for depression among adult cancer patients, as identified across the 47 included narrative reviews. As shown in the graph, risk factorswithin the “genetic-biological” domain were the most commonly analyzed, with r “pro-inflammatory cytokines” emerging as the most extensively studied factor. It also appears that protective factors (indicated in green) hwere comparatively less frequently investigated. Among these, social support stands out as the most consistently examined protective factor.

The figure shows the main DAFs examined in narrative reviews, with the number of studies supporting each association and the direction of the association (risk vs. protective).

### Prospective DAFs

In summary, when considering only prospective studies, the following DAFs met the criteria of PCS ≥75% and ≥5 studies: high social support (8/9 studies, 88,90%), cancer-related physical symptoms (5/6 studies, 83,30%), time dedicated to patient communication (5/5 studies, 100%) and history of depression (8/10 studies, 80,00%).

Additional DAFs with PCS ≥75%, though supported by ≤5 syudies included: rumination (3/3 studies, 100%), anxiety (3/3 studies, 100%), distress (1/1 studies, 100%), fatigue (1/1 studies, 100%), spirituality (3/3 studies, 100%), self-esteem (1/1 studies, 100%), self-humor (1/1 studies, 100%), communication dysfunctions (1/1 studies, 100%), malnutrition (2/2 studies, 100%), neck disability (1/1 studies, 100%), low educational level (1/1 studies, 100%), lower income (1/1 studies, 100%), longer time since surgery (1/1 studies, 100%), and lobectomy (1/1 studies, 100%).

### Meta-analyses

The following significant results emerged: IL-6 in 31 primary studies (SMD 0.59; 95% CI, 0.35–0.82), TNF-α in 21 primary studies (SMD 0.73; 95% CI, 0.35–1.11), and CRP in 10 studies (SMD 0.57; 95% CI, 0.27–0.87). Two meta-analyses reported a significant association with ADT therapy: the first (Siebert et al. 2020) found an increased risk of depression among prostate cancer patients treated with ADT (HR = 1.51; 95% CI, 1.34–1.69), while the second (Nead et al. 2017) reported a similar increase in risk of depression (RR = 1.41; 95% CI, 1.18–1.70).

One meta-analysis (Padmalatha et al. 2021) showed an increased risk of depression in women undergoing total mastectomy without breast reconstruction (RR = 1.36, 95% CI: 1.11–1.65), while chemotherapy was significantly associated with depression in 3 primary studies (SMD 0.28, 95% CI: 0.06–0.5). Additional significant associations were observed for: total mastectomy (9 primary studies; RR 1.42, 95% CI: 1.06–1.89) and brain tumor location (overall prevalence among cancer survivors was 22%).

Non-significant or inconsistent findings were reported for the serotonin-transporter-linked polymorphic region (5-HTTLPR) (6 primary studies) and for comparisons between total mastectomy (TM), breast conserving therapy (BCS) and Breast Reconstruction (BR), with no statistically significant differences observed across 16 primary studies).

### Risk of bias

According to the JBI Critical Appraisal Checklist (Aromataris et al. 2024; Hilton 2024), the methodological quality of most included systematic reviews was high (*n* = 20/26), while only 2 out of 26 studies were rated as having a high risk of bias score (Supplementary Table S2). The most common issues concerned insufficient reprtong or inadequate assessment of publication bias (Table S2, item 9). Additions critical flaws included the absence and inadequacy methodological appraisal and the lack of independent assessment by two or more reviewers independently (Table S2, questions 5 and 6).

### Methodological appraisal

Eighteen out of twenty-six systematic reviews (69.23%) included a formal methodological appraisal, and all were rated as high quality according to the JBI Critical Appraisal Checklist. Among these, 8 explicitly reported the number of primary studies rated as high quality; overall, 50.42% of primary studies were classified as high quality.

Eight systematic reviews (8/26) as well as all narrative reviews did not formally assess the risk of bias of included primary studies. Even when such assessments were conducted, different tools were used (e.g., GRADE, ROBIS), contributing to methodological variability.

## Discussion

We reviewed secondary literature on factors associated with depression (DAFs) among individuals with cancer. Cross-sectional studies consistently identified associations between depression and several sociodemographic factors (e.g., high social support, being unmarried), inflammatory markers (IL-6, TNF-α, CRP), psychological variables (e.g., history of depression, anxiety, rumination, distress, high levels of hope), and certain somatic characteristics (e.g., fatigue, low functional status, malnutrition). Longitudinal studies confirmed key predictiors of depression onset, inlcuding malnutrition, history of depression, anxiety, rumination, distress, fatigue and high social support, and identified additional candidate DAFs, such as cancer-related physical symptoms, physical comorbidities, time dedicated to patient communication, spirituality, self-esteem and self-humor, longer time since surgery and low educational level.

Several DAFs identified in this umbrella review – low educational level, being unmarried, IL-6, CRP, TNF-α, anxiety, rumination, distress, history of depression, fatigue, low functional status, physical comorbidities and malnutrition – are also consistently associated with both the onset and the course of depression in non-cancer populations (Rakel 1999; Lorant et al. 2003; Anagnostopoulos et al. 2004; Honda and Goodwin 2004; Laird et al. 2009; Santini et al. 2015; Gariépy et al. 2016; Grassi et al. 2017; Lund et al. 2018; Malhi and Mann 2018; Belvederi Murri et al. 2022; Li et al. 2023b). High social support was protective, whereas being unmarried increased risk, indicating the role of interpersonal networks in depression, across both general and oncological populations (Li et al. 2023a), (Grassi et al. 1997; Yan et al. 2011). Findings on the 5-HTTLPR polymorphism remain inconsistent: while meta-analysis in the general-population suggest possible moderation of depression risk under stress, oncology-specific evidence, including a meta-analysis of cancer patients, does not support an association (Grassi et al. 2010; Karg et al. 2011; Suppli et al. 2015; Culverhouse et al. 2018). We also identified factors such as cancer-related physical symptoms, time dedicated to communication , hope , and pain intensity, which reflect contextual influences specific to oncology. High hope has been reported as protective, whereas hopelessness is associated with poorer adaptation and increased depression risk, indicating a role for motivational and goal-oriented coping in cancer care (Anagnostopoulos et al. 2004; Grassi et al. 2017).

Some well-established DAFs in the general population did not emerge consistently in our umbrella review. Female gender, although widely recognized as a risk factor outside oncology (Kuehner 2017; Caruso et al. 2017b), was significant in only 36% of included studies (Ajmera et al. 2025). Coping strategies, another relevant factor in the general population (Almeida et al. 2021), were relatively underexplored in oncology, with fewer than five studies examining it, all reporting significant associations, but none with a longitudinal design. This highlights the need to conduct further research, since assessment instruments exist to capture the multifactorial nature of coping – which is strictly related to resilience and understudied (Tamura et al. 2021) – and emotion regulation (Trudel-Fitzgerald et al. 2024). Positive coping has been recently reframed within the construct of post-traumatic growth (PTG; Zoellner and Maercker 2006).

Delay recognition and management of depression in cancer patients impair quality of life, reduce treatment adherence, hinder rehabilitation and may even increase morbidity and mortality (Härter et al. 2007; McFarland et al. 2021; Grassi et al. 2025). Identifying DAFs, pending confirmation of causal roles, may inform risk prediction models and guide targeted prevention (Van Smeden et al. 2021; Erim et al. 2019). Similar reviews have supported the selection of predictors for the development of models to stratify older patients for early detection of depression (Belvederi Murri et al. 2022). Greater emphasis on modifiable factors may clarify their role to support preventive strategies (Irwin et al. 2022); however clinical trials are required to establish which interventions are effective in this specific population.

Depression in oncology is heterogeneous, with distinct patterns linked to biological and social DAFs that may reflect different pathways to onset (Ostergaard et al. 2013; Gaspersz et al. 2018; Beijers et al. 2019). This heterogeneity complicates both diagnosis and treatment, underscoring the need for stratified approaches within personalized medicine. Our findings may also inform research on related constructs such as PTG.

Current theoretical models address only parts of this complexity (Haslbeck et al. 2021). Sociodemographic factors such as social support and marital status influence both risk and protection, with evidence showing that social resources reduce distress and improve quality of life (Nipp et al. 2016; Chen et al. 2023; Aromataris et al. 2024). Psychological factors, including prior depression, anxiety, and distress increase vulnerability, whereas hope enhances resilience and coping (Beck 2008; Caruso et al. 2017b). Biological mechanisms highlight immune–inflammatory processes and alterations in the kynurenine pathway as plausible links beteween cancer, fatigue, and depression (Dantzer et al. 2008; Sforzini et al. 2019; Belvederi Murri et al. 2020; Zerbinati et al. 2021; Nusslock et al. 2024). However, integrative explanatory models remain limited (Haslbeck et al. 2021). Emerging frameworks such as network theory, which conceptualizes depression as a dynamic system of interconnected symptoms, may help integrate influences across multiple levels, from social to biological (Cramer et al. 2016; Belvederi Murri et al. 2020, 2023), (Bickel et al. 2024).

The main strength of this review lies in its comprehensive search strategy and the combined use of quantitative and qualitative approaches to assess evidence credibility. To our knowledge, this is the first umbrella review to synthesize DAFs across all domains, while evaluating both the strengths and consistency of evidence using standardized criteria (Aromataris et al. 2024). Alythough the inclusion of diverse cancer types increases heterogeneity, it also provides a broad overview of potential DAFs. The findings of this umbrella review should be considered in light of the limitations of secondary and primary literature that were included. First, there was a predominance of narrative over systematic reviews, along with an relatively low proportion of prospective primary studies (approximately one third of the total number). This limits the representativeness of the findings and the possibility of drawing any conclusions regarding causality. In addition, only 31% of systematic reviews assessed publication bias in primary studies. Despite the breadth of included literature, publication bias remains a key concern of our review, partly due to the exclusion of studies without accessible full text. Considering publication bias, primary studies on biological factors may be particularly susceptible to the file-drawer problem (Ioannidis 2005), whereas studies on sociodemographic variables, althoug less resource-intensive, may still be affected by overadjustment or inadequate modeling of underlying causal relationships (Cinelli et al. 2024). Further limitations include heterogeneity in populations, definitions of risk factor , cancer sites, and the non-systematic consideration of attrition, all which may reduce generalizability. In partiular, the relative overrepresentation of specific cancer sites (e.g., breast or prostate cancer) may have introduced additional bias.

## Conclusion

Our umbrella review identified several factors reliably associated with depression in individual with cancer, including sociodemographic factors (e.g., high social support, being unmarried), inflammatory markers (IL-6, TNF-α, CRP), psychological factors (e.g., history of depression, anxiety, rumination, distress, high levels of hope), and certain somatic factors (e.g., fatigue, low functional status, malnutrition). Our findings may inform strategies for screening or early identification of depression in oncological populations. Further research, particularly longitudinal studies, is needed to clarify the role of genetic polymorphisms, specific cancer-related factors, and oncological treatments, for which current evidence remains largely inconsistent. Given the high burden of depression among people with cancer, personalized and preventive approaches may help address the complex mental health challenges faced by this population.

## Supporting information

10.1017/S1478951526102247.sm001 Zaccagnino et al. supplementary material Zaccagnino et al. supplementary material

## Data Availability

All data generated or analyzed during this study are included in this article. Further enquiries can be directed to the corresponding author.

## Supplementary material

The supplementary material for this article can be found at https://doi.org/10.1017/S1478951526102247.
