# Supplementary material for: Factors associated with depression among people with cancer: Systematic umbrella review
Source: Palliat Support Care. 2026 May 15;24:e146. doi: 10.1017/S1478951526102247 (PMC13202400; doi:10.1017/S1478951526102247)
Supplement: Zaccagnino et al. supplementary material [file S1478951526102247sup001.docx]

**Supplementary material**

**S1. Supplementary methods**

S1.2 Search strings

Pubmed: (depression AND cancer) AND (review OR meta-analysis).

Filters: publication date, text availability, English language, and adults (≥18 years)

**Table S1. Reasons for exclusion**

The table reports the list of excluded studies. For each secondary literature article, the following information is provided: author, year, full title, stage of the process in which the exclusion occurred, and reason for exclusion.

| PROGRESSIVE NUMBER | AUTHOR / YEAR | STUDY TITLE | WHEN | WHY |
| --- | --- | --- | --- | --- |
| 1 | Pereira da Silva Juver, Verçosa 2008 | Depression in Patients with Advanced Cancer and Pain | Records removed before screening | other language |
| 2 | Reynaert 2000 | Psychogenesis of cancer: between myths, misuses and reality | Records removed before screening | other language |
| 3 | Ronson 2007 | Neurotrophic theories of stress and neurobiology of antidepressants: applications in psycho-oncology | Records removed before screening | other language |
|  |  |  |  |  |
| 1 | Bakula et all.2019 | The Relationship Between Parent and Child Distress in Pediatric Cancer: A Meta-Analysis | Title/Abstract | <18 years |
| 2 | Benson et al. 2022 | Psychiatric Considerations in Pediatric Patients With Brain Tumors | Title/Abstract | <18 years |
| 3 | Burstein 2007 | Cognitive side-effects of adjuvant treatments | Title/Abstract | No review |
| 4 | Grusdat et al. 2022 | Routine cancer treatments and their impact on physical function, symptoms of cancer‑related fatigue, anxiety, and depression | Title/Abstract | Prospective observational study |
| 5 | Henry et al. 2008 | Drug Interactions and Pharmacogenomics in the Treatment forBreast Cancer and Depression | Title/Abstract | Case report |
| 6 | Lampic, Sjodén 2000 | Patient and Staff Perceptions of Cancer Patients' Psychological Concerns and Need | Title/Abstract | Off topic |
| 7 | Lang et al. 2015 | The Age Conundrum: A Scoping Review of Younger Age or Adolescent and Young Adult as a Risk Factor for Clinical Distress, Depression, or Anxiety in Cancer | Title/Abstract | <18 years |
| 8 | McNicol et al. 2003 | Management of Opioid Side Effects in Cancer-Related and Chronic Noncancer Pain: A Systematic Review | Title/Abstract | Not relevant |
| 9 | Narendran 2018 | Inflammatory cytokines and depression in children with cancer: a review of the literature | Title/Abstract | <18 years |
| 10 | Oh 2016 | Predictors of cognitive decline in people with cancer undergoing chemotherapy | Title/Abstract | Cross sectional study |
| 11 | Packman 2010 | Psychological effects of hematopoietic SCT on pediatric patients,siblings and parents: a review | Title/Abstract | <18 years |
| 12 | Spiegel, Kato 1996 | Psychosocial influences on cancer incidence and progression | Title/Abstract | Off topic |
| 13 | Thaduri et al. 2022 | Financial toxicity and mental well‐being of the oral cancer survivors residing in a developing country in the era of COVID 19 pandemic – A cross‐sectional study | Title/Abstract | Cross sectional study |
| 14 | Vadaparampil et al. 2005 | Measurement of psychological factors associated with genetic testing for hereditary breast, ovarian and colon cancers | Title/Abstract | Off topic |
| 15 | Yuce 2021 | Psychological distress and the association with unmet needs and symptom burden in outpatients: a cross-sectional study | Title/Abstract | Cross-sectional study |
|  |  |  |  |  |
| 1 | Bergantin 2020 | A Hypothesis for the Relationship between Depression and Cancer: Role of Ca2+/cAMP Signalling | Full-text | Reports not retrieved |
| 2 | Braczkowski 1999 | Depression as a risk factor for cancer--is it still a hypothesis or a proven fact? | Full-text | Reports not retrieved |
| 3 | Conti et al. 2011 | Relationship between cancer and psychology: an updated history | Full-text | Reports not retrieved |
| 4 | Estrella Castillo 2020 | Scientific evidence of the relation between breast cancer and depression: systematic review | Full-text | Reports not retrieved |
| 5 | Ginter, Simko 2010 | Polyunsaturated fatty acids n-3: new data on heart disease, cancer, immune resistance and mental depression | Full-text | Reports not retrieved |
| 6 | Illman 2005 | Are inflammatory cytokines the common link between cancer-associated cachexia and depression? | Full-text | Reports not retrieved |
| 7 | Moussas - 2012 | Psychological and psychiatric problems in cancer patients: relationship to the localization of the disease | Full-text | Reports not retrieved |
| 8 | Nothdurfter 2007 | The diagnosis is cancer--and then depression? | Full-text | Reports not retrieved |
|  |  |  |  |  |
| 1 | Alexander et al. 2007 | Arthralgias, bodily aches and pains and somatic complaints in midlife women etiology, pathophysiology and differential diagnosis | Full text screening | Risk factors are not considered |
| 2 | Antoni et al. 2006 | The influence of bio-behavioural factors on tumour biology: pathways and mechanisms | Full text screening | Depression as risk factor for cancer development and progression |
| 3 | Armaiz-Pena et al. 2009 | Neuroendocrine modulation of cancer progression | Full text screening | Depression as risk factor for cancer development and progression |
| 4 | Bleiker et all.1999 | Psycosocial factorsin the etiology of breast cancer: review of a popular link | Full text screening | Scoping review |
| 5 | Brewer 2008 | Behavioral Genetics of the Depression/Cancer Correlation: A Look at the Ras Oncogene Family and the ‘Cerebral Diabetes Paradigm*’* | Full text screening | Correlation between depression and later cancer onset |
| 6 | Chan et al. 2012 | Clinically-- relevant anticancer antidepressant drug interactions | Full text screening | Specific risk factors for depression are not considered |
| 7 | Chapman et al. 2005 | The Vital Link Between Chronic Disease and Depressive Disorders | Full text screening | Specific risk factors for depression are not considered |
| 8 | Colucci, D'Amato 2020 | Neurotrophic Factor BDNF, Physiological Functions and Therapeutic Potential in Depression, Neurodegeneration and Brain Cancer | Full text screening | Brain gliomas, depression and Alzheimer disease separately |
| 9 | Cuevas et al. 2019 | Placing prostate cancer disparities within a psychosocial context: Challenges and opportunities for future research | Full text screening | Depression as a risk factor for (prostate) cancer |
| 10 | Currier e Nemeroff 2014 | Depression as a risk factor for cancer from pathophysiological advances to treatment implications | Full text screening | Depression as a risk factor for cancer |
| 11 | Davis et al. 2006 | Management of Fatigue in Cancer Patients | Full text screening | Depression as contributory factor for fatigue in cancer patients |
| 12 | Ebede et al. 2017 | Cancer-Related Fatigue in Cancer Survivorship | Full text screening | Depression as contributory factor for fatigue in cancer patients |
| 13 | Ellis, Tattersall 1999 | How should doctors communicate the diagnosis of cancer to patients? | Full text screening | This article is more a reflection on good medical practice and intend to work as a vademecum for psycho-oncologists more than analyze the good medical communication as a protective factor |
| 14 | Fortin et al. 2021 | The mental health impacts of receiving a breast cancer diagnosis: A meta-analysis | Full text screening | Meta-analyisis of prevalence |
| 15 | Froes Brandao et al. 2015 | Prolactin and Breast Cancer: The Need to Avoid Undertreatment of Serious Psychiatric Illnesses in Breast Cancer Patients: A Review | Full text screening | The review doesn't analyse the role of PRL in the development of depression in breast cancer patients. |
| 16 | Ginter, Simko 2010 | Polyunsaturated fatty acids n-3: new data on heart disease, cancer, immune resistance and mental depression | Full text screening | Cancer and depression separately |
| 17 | Huang et al. 2022 | Risk factors for cancer‑related fatigue in patients with colorectal cancer: a systematic review and meta‑analysis | Full text screening | Depression as a risk factor for fatigue in (colorectal) cancer patients |
| 18 | Husson et al. 2011 | The relation between information provision and health related quality of life, anxiety and depression among cancer survivors: a systematic review | Full text screening | Specific risk factors for depression are not considered |
| 19 | Irwin, Miller 2007 | Depressive disorders and immunity, 20 years of progress and discovery | Full text screening | Risk factors are not considered |
| 20 | Izard et al. 2019 | Androgen Deprivation Therapy and Mental Health: Impact on Depression and Cognition | Full text screening | Mini review |
| 21 | Kallay 2022 | On top of that all, now Covid-19 too: a scoping review of specificities and correlates of fear of cancer recurrence in breast cancer patients during COVID-19 | Full text screening | Specific risk factors for depression are not considered |
| 22 | Kiecolt-Glaser et al. 1999 | Psychoneuroimmunology and cancer, fact or fiction | Full text screening | Risk factors are not considered |
| 23 | Kirkova et al. 2011 | Cancer Symptom Clusters: Clinical and Research Methodology | Full text screening | Depression is considered in a cluster of different symptoms |
| 24 | Lutgendorf et al. 2010 | Host Factors and Cancer Progression: Biobehavioral Signaling Pathways and Interventions | Full text screening | Depression as risk factor for cancer progression |
| 25 | McCorkle et al. 2003 | The Silent Killer Psychological Issues in Ovarian Cancer | Full text screening | Specific risk factors for depression are not considered |
| 26 | Moyad, Pienta 2002 | MIND-BODY EFFECT: INSULINLIKE GROWTH FACTOR–1; CLINICAL DEPRESSION; AND BREAST, PROSTATE, AND OTHER CANCER RISK—AN UNMEASURED AND MASKED MEDIATOR OF POTENTIAL SIGNIFICANCE? | Full text screening | Depression as a risk factor for cancer development |
| 27 | Novy et all. 2014 | The bio social model in cancer pain | Full text screening | Specific risk factors for depression are not considered |
| 28 | Reiche 2004 | Stress, depression, the immune system and cancer | Full text screening | Depression as a risk factor for cancer |
| 29 | Rothen 2013 | Body image in patients with head and neck cancer: a review of the literature | Full text screening | Risk factors are not considered |
| 30 | Santos, Pyter 2018 | Neuroimmunology of behavioral comorbidities associated with cancer and cancer treatments | Full text screening | Only animal studies included |
| 31 | Senra, McPherson 2021 | Depression in disabling medical conditions, current perspective | Full text screening | Specific risk factors for depression are not considered |
| 32 | Schneider 2010 | Pre-intervention distress moderates the efficacy of psychosocial treatment for cancer patients: a meta-analysis | Full text screening | Meta-analyisis of prevalence |
| 33 | Valentine 2001 | Cognitive and Mood Disturbance as Causes and Symptoms of Fatigue in Cancer Patients | Full text screening | Risk factors are not considered |
| 34 | Werneke 2007 | Risk management of nutritional supplements in chronic illness:the implications for the care of cancer and depression | Full text screening | Risk factors are not considered |
| 35 | Wu et al. 2023 | Investigating the relationship between depression and breast cancer: observational and genetic analyses | Full text screening | Depression as risk factor for cancer |
| 36 | Ye et al. 2022 | Crosstalk between Depression and Breast Cancer via Hepatic Epoxide Metabolism: A Central Comorbidity Mechanism | Full text screening | Depression as a risk factor for cancer development |

**S2. Supplementary results**

**Table S2. JBI assessment of the included systematic reviews and meta-analyses**

The table presents the appraisal of methodological quality and risk of bias of this umbrella review, carried out using the Joanna Briggs Institute (JBI) Critical Appraisal Checklist for Systematic Reviews and Research Syntheses. All 26 narrative reviews and meta-analyses included in the umbrella review were assessed with this instrument. The checklist consists of 11 items: the first 9 address potential sources of bias and methodological rigor, while the last 2 focus on impact and implications for future research. Each item can be rated as “yes,” “no,” “unclear,” or “not applicable (NA).” The overall appraisal outcome is reported in the last column.

| JBI questions | 1. Is the review question clearly and explicitly stated? | 2. Were the inclusion criteria appropriate for the review question? | 3. Was the search strategy appropriate? | 4. Were the sources and resources used to search for studies adequate? | 5. Were the criteria for appraising studies appropriate? | 6. Was critical appraisal conducted by two or more reviewers independently? | 7. Were there methods to minimize errors in data extraction? | 8. Were the methods used to combine studies appropriate? | 9. Was the likelihood of publication bias assessed? | 10. Were recommendations for policy and/or practice supported by the reported data? | 11. Were the specific directives for new research appropriate? | TOTAL |
| --- | --- | --- | --- | --- | --- | --- | --- | --- | --- | --- | --- | --- |
| Alexander et al., 2023 | YES | YES | YES | YES | NO | NA | YES | YES | NO | YES | YES | 8/11 |
| Alwahibi et al., 2022 | YES | YES | YES | YES | NO | NA | NO | YES | NO | YES | NO | 6/11 |
| Ayubi et al., 2021 | YES | YES | YES | YES | NO | NA | NO | YES | NO | YES | YES | 7/11 |
| Batra et al., 2020 | YES | YES | YES | YES | YES | YES | YES | YES | YES | YES | YES | 11/11 |
| Beck et al., 2023 | YES | YES | YES | YES | YES | YES | YES | YES | NO | YES | YES | 10/11 |
| Bellardita et al., 2015 | YES | YES | YES | YES | NO | NA | YES | YES | NO | YES | YES | 8/11 |
| Chair et al., 2022 | YES | YES | YES | YES | YES | YES | YES | YES | NO | YES | YES | 10/11 |
| Freitas and Campos, 2019 | YES | NO | NO | NO | NO | NA | NO | YES | NO | YES | YES | 4/11 |
| Kitashita and Suzuki, 2023 | YES | YES | YES | YES | YES | YES | YES | YES | NO | YES | YES | 10/11 |
| Korsten et al., 2019 | YES | YES | YES | YES | YES | YES | YES | YES | NO | YES | YES | 10/11 |
| Ibrahim et al., 2021 | YES | YES | YES | YES | YES | YES | YES | YES | YES | YES | YES | 11/11 |
| Laird et al., 2009 | YES | YES | YES | YES | YES | YES | NO | YES | NO | YES | YES | 9/11 |
| Lee et al., 2023 | YES | YES | YES | YES | YES | YES | YES | YES | YES | YES | YES | 11/11 |
| McFarland et al., 2022 | YES | YES | YES | YES | YES | YES | YES | YES | YES | YES | YES | 11/11 |
| Nead et al., 2017 | YES | YES | YES | YES | YES | YES | YES | YES | YES | YES | YES | 11/11 |
| Padmalatha et al., 2021 | YES | YES | YES | YES | YES | YES | YES | YES | YES | YES | YES | 11/11 |
| Patton et al., 2020 | YES | YES | YES | YES | YES | YES | YES | YES | NO | YES | YES | 10/11 |
| Pop et al., 2017 | YES | NO | NO | NO | NO | NA | NO | YES | NO | YES | NO | 3/11 |
| Riedl and Schüßler, 2022 | YES | YES | YES | YES | YES | UNCLEAR | YES | YES | NO | YES | YES | 9/11 |
| Sforzini et al., 2019 | YES | YES | YES | YES | NO | NA | NO | YES | NO | YES | YES | 7/11 |
| Siebert et al., 2020 | YES | YES | YES | YES | YES | UNCLEAR | NO | YES | NO | YES | YES | 8/11 |
| Smith et al., 2018 | YES | YES | YES | YES | YES | YES | YES | YES | NO | YES | YES | 10/11 |
| Suppli et al., 2015 | YES | YES | YES | YES | NO | NA | UNCLEAR | YES | NO | YES | YES | 7/11 |
| Szabados et al., 2023 | YES | YES | YES | YES | YES | YES | YES | YES | YES | YES | YES | 11/11 |
| Wen et al., 2019 | YES | YES | YES | YES | YES | YES | YES | YES | NO | YES | YES | 10/11 |
| Zhang et al., 2018 | YES | YES | YES | YES | YES | YES | YES | YES | YES | YES | YES | 11/11 |

**Table S3. Narrative reviews characteristics**

The table presents the list of narrative reviews. For each review, the following data are reported: author, publication year, number of studies, study setting, and name of the DAFs.

| **Citation** | **Study design** | **N of studies** | **Study settings, partecipant eligibility** | **Associated factors names** |
| --- | --- | --- | --- | --- |
| **Archer et al. (2008)** | Narrative review | 18 | Adults, head and neck cancer | Abnormalities of the HPAA |
| **Ahmad et al. (2021)** | Narrative review | 86 | Adults, no specific type of cancer reported | Pro-inflammatory cytokines, dysregulated HPA-axis activity |
| **Aldea et al. (2014)** | Narrative review | 73 | Adults, no specific type of cancer reported | Pro-inflammatory cytokines (IL-6, TNF-a, CRP, IL-1ra), dysregulated HPA-axis activity, increased level of IDO or TDO |
| **Barnes et al. (2018)** | Narrative review | 31 | Adults, no specific type of cancer reported | TRP degradation (overexpression of IDO), Antibody production against 5-HT receptors |
| **Barreto et al. (2018)** | Narrative review | 16 | Adults, ovarian cancer | Proinflammatory cytokines (IL-1β, IL-6, TNF-α, and IL-10), IDO and the TRYCAT pathway |
| **Bates et al. (2022)** | Narrative review | 9 | Adults, no specific type of cancer reported | Long-term prescription opioid therapy |
| **Borsellino, Young (2011)** | Narrative review | 12 | Adults, no specific type of cancer reported | Hair loss, anticipatory coping of hair loss |
| **Bortolato et al. (2016)** | Narrative review | 9 | Adults, no specific type of cancer reported | Pro-inflammatory cytokines (IL-6, IL-8, IL-1, TNF-, sIL-2R, CRP) |
| **Brescia F. J. (2004)** | Narrative review | 5 | Adults, pancreatic cancer | Pain, chemotherapy |
| **Capuron e Dantzer (2003)** | Narrative review | 43 | Adults, no specific type of cancer reported | Pro-inflammatory cytokines, dysregulated HPA-axis activity, increased level of IDO |
| **Dantzer et al. (2008)** | Narrative review | 27 | Adults, no specific type of cancer reported | Pro-inflammatory cytokines (IL-1α and IL-1β, TNF-α, IL-6), increased level of IDO |
| **Donovan et al. (2015)** | Narrative review | 31 | Adults, prostate cancer | Androgen deprivation therapy, to be married/in a relationship |
| **Fagundes et al. (2015)** | Narrative review | 72 | Adults, breast cancer | Pro-inflammatory cytokines, dysregulation of the hypothalamic-pituitary-adrenal (HPA) axis, stressful life experiences, lower socioeconomic position, social support, physical activity |
| **Fasano et al. (2020)** | Narrative review | 101 | Adults, breast cancer | Optimism, coping strategies (seeking social support and emotion-focused coping), negative religious coping, resilience |
| **Feldman and Corn (2023)** | Narrative review | 45 | Adults, no specific type of cancer reported | Hope |
| **Fervaha et al. (2019)** | Narrative review | 10 | Adults, prostate cancer | Cancer stage, erectile dysfunction, urinary/bowel symptoms, testosterone levels, race, age, pain, personality characteristics, cognitive biases - catastrophizing, coping strategies, personal psychiatric history, socioeconomic status, access to/ vicinity of specialist care, family and social support |
| **Gagliese et al. (2007)** | Narrative review | 4 | Adults, no specific type of cancer reported | Pain |
| **Green, Austin (1993)** | Narrative review | 68 | Adults, pancreatic cancer | ACHT and CRH, PTH and PTH-Iike factor, TRH, Glucagon, serotonin deficiency |
| **Greenberg (2004)** | Narrative review | 22 | Adults, no specific type of cancer reported | Past history of depression, social support, family history of depression, psychological resilience, effective communication with healthcare providers, anxiety disorder, access to mental health care, substance abuse, engagement in treatment protocols, medical symptoms and situational distress, cancer medications |
| **Grusdat et al. (2022)** | Narrative review | 79 | Adults, breast cancer | Early stage cancer diagnosis |
| **Horrobin, Bennett (1999)** | Narrative review | 6 | Adults, no specific type of cancer reported | Cytokines: Interferon and Interleukin-2 |
| **Jarrin Jara (2020)** | Narrative review | 28 | Adults, pancreatic cancer | Pro-inflammatory cytokines (IL-6) |
| **Kissane (2009)** | Narrative review | 53 | Adults, breast cancer | Social disadvantage, social support, hopelessness, psychoterapy interventions, coping interventions, co-morbid conditions, access to preventive screening, psychological response to cancer treatment |
| **Kurz et al. (2011)** | Narrative review | 25 | Adults, no specific type of cancer reported | Immune-mediated tryptophan degradation |
| **Lacina et al. (2019)** | Narrative review | 67 | Adults, no specific type of cancer reported | Pro-inflammatory cytokines (IL-6, IL-6r) |
| **Lanser et al. (2020)** | Narrative review | 93 | Adults, no specific type of cancer reported | Increased level of IDO, anti-infiammatory diet, increased kynurenine to tryptophan ratio (Kyn/Trp), physical activity, age, gender, race, coping style, low psychosocial support, high psychosocial support, increased neopterin concentrations, pro-inflammatory cytokines (IFN-gamma) |
| **Leonard (2000)** | Narrative review | 54 | Adults, no specific type of cancer reported | Pro-inflammatory cytokines, dysregulated HPA-axis activity |
| **Maggi et al. (2018)** | Narrative review | 18 | Adults, prostate cancer | Urinary symptoms related to incontinence |
| **Mampay et al. (2021)** | Narrative review | 84 | Adults, no CNS cancers | Tumour-induced activation of the immune system, blood–brain barrier breakdown, chronic neuroinflammation |
| **McFarland et al. (2021)** | Narrative review | 14 | Adults, no specific type of cancer reported | Pro-inflammatory cytokines (IL-6, IFN-gamma, TNFa, IL-1b), increased level of IDO or TDO |
| **Michoglou et al. (2023)** | Narrative review | 15 | Adults, pancreatic cancer | Increased urinary excretion of 5-hydroxyindolaceetic acid (5-HIAA), high concentration of 5-hydroxytryptophne (5-HTP) or 5-hydroxytryptamine (5-HT) |
| **Myers et al. (2010)** | Narrative review | 14 | Adults, renal cell cancer and metastatic melanoma | Exogenous administration of IL-2, antidepressants prophylactic therapy (paroxetine), elevated levels of IL-6, exogenous administration of IL-2, IFNa, IFNb |
| **Numakawa et al. (2014)** | Narrative review | 43 | Adults, ovarian cancer and breast cancer | Treatment with IFN-α, treatment with tamoxifene |
| **Parker et al. (2017)** | Narrative review | 4 | Adults, pancreatic cancer | Increased inflammatory markers (IL6, IL18, TNFa) |
| **Pollak and Yirmiya (2002)** | Narrative review | 35 | Adults, no specific type of cancer reported | Exogenous administration of IL-2, IFNa, TNFa, increased levels of IL-6, IL-8, IL-10 with decreased levels of serum dipeptidyl peptidase IV (correlated to immunotherapy), elevated levels of serum neopterin, decreased levels of 5HT and tryptophan |
| **Politynska et al. (2022)** | Narrative review | 50 | Adults, no specific type of cancer reported | Chronic stress, kynurenine pathway alterations, glutamatergic excitotoxicity |
| **Reyes-Gibbi (2012)** | Narrative review | 23 | Adults, no specific type of cancer reported | Increased levels of IL-6, cancer type, cancer stage, age, gender, marital status, race |
| **Schiepers et al. (2005)** | Narrative review | 38 | Adults, no specific type of cancer reported | Cancer therapies (treatment with IL-2, TNFa, IFNa), pre-treatment with cytokine synthesis inhibitors and cytokine antagonists or by cytokine gene manipulation, immune dysregulation |
| **Schrepf et al. (2015)** | Narrative review | 71 | Adults, no specific type of cancer reported | Cytokine-based treatment of cancers (i.e. INFa), elevated plasma levels of IL-6, soluble IL-6 receptors, CRP and abnormal dexamethasone suppression tests, elevated TNFa in serum, elevated solbule TNF receptors levels, high Glasgow Prognostic Score, lower kynurenic acid/tryptophan ratio associated with worse symptoms of depression |
| **Seruga et al. (2008)** | Narrative review | 60 | Adults, no specific type of cancer reported | High levels of IL-6, decreased levels of circulating tryptophan in patients undergoing therapy with IL-2 or TNFa |
| **Sharpley et al. (2020)** | Narrative review | 51 | Adults, prostate cancer | Initial diagnosis, information, psychological resilience, biopsy, uncertain outcome, androgen deprivation therapy, urinary incontinence, suicidal thoughts |
| **Sotelo et al. (2014)** | Narrative review | 60 | Adults, no specific type of cancer reported | Innate immune mediators, dysregulated patterns of cortisol secretion, chronic high dose of IFN-α, pro-inflammatory cytokines: tumour necrosis factor α (TNF- α), interleukin 1 (IL-1), and interleukin 6 (IL-6) |
| **Spoletini et al. (2009)** | Narrative review | 21 | Adults, no specific type of cancer reported | Pain, proinflammatory cytokine (TNF- α, IL-1 and IL-6) |
| **Theobald (2004)** | Narrative review | 4 | Adults, no specific type of cancer reported | Insomnia |
| **Traish et al. (2014)** | Narrative review | 8 | Adults, prostate cancer | Finasteride treatment |
| **Valentine (2003)** | Narrative review | 4 | Adults, no specific type of cancer reported | Pain |
| **Young and Singh (2018)** | Narrative review | 68 | Adults, no specific type of cancer reported | High plasma concentration of IL-6, microglia activation, hyperactivation of HPA axis, glutamate exitotoxicity |

**Figure S1. DAFs in Primary Studies: ten Most Frequently Investigated**


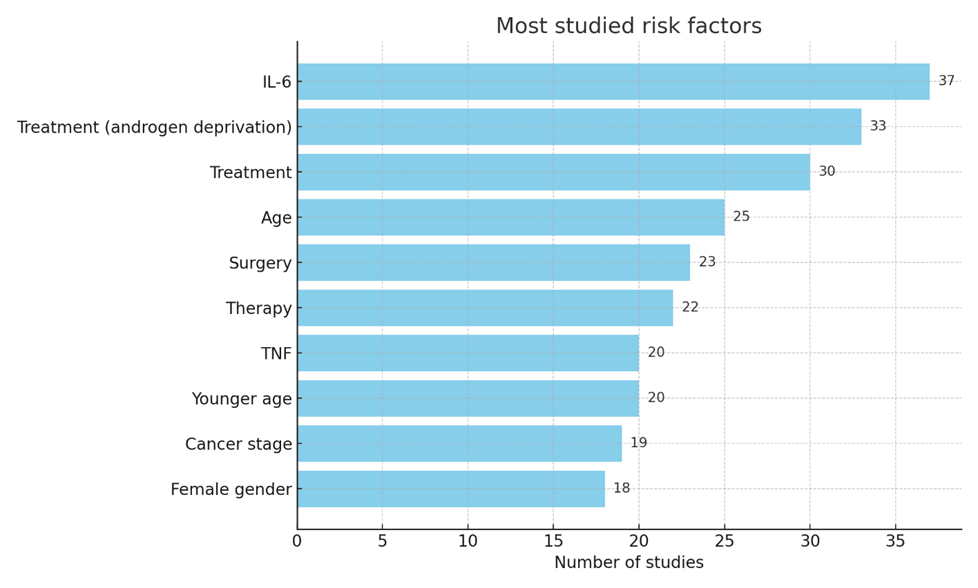


**Figure S2. Association between biological-genetic factors and depression**


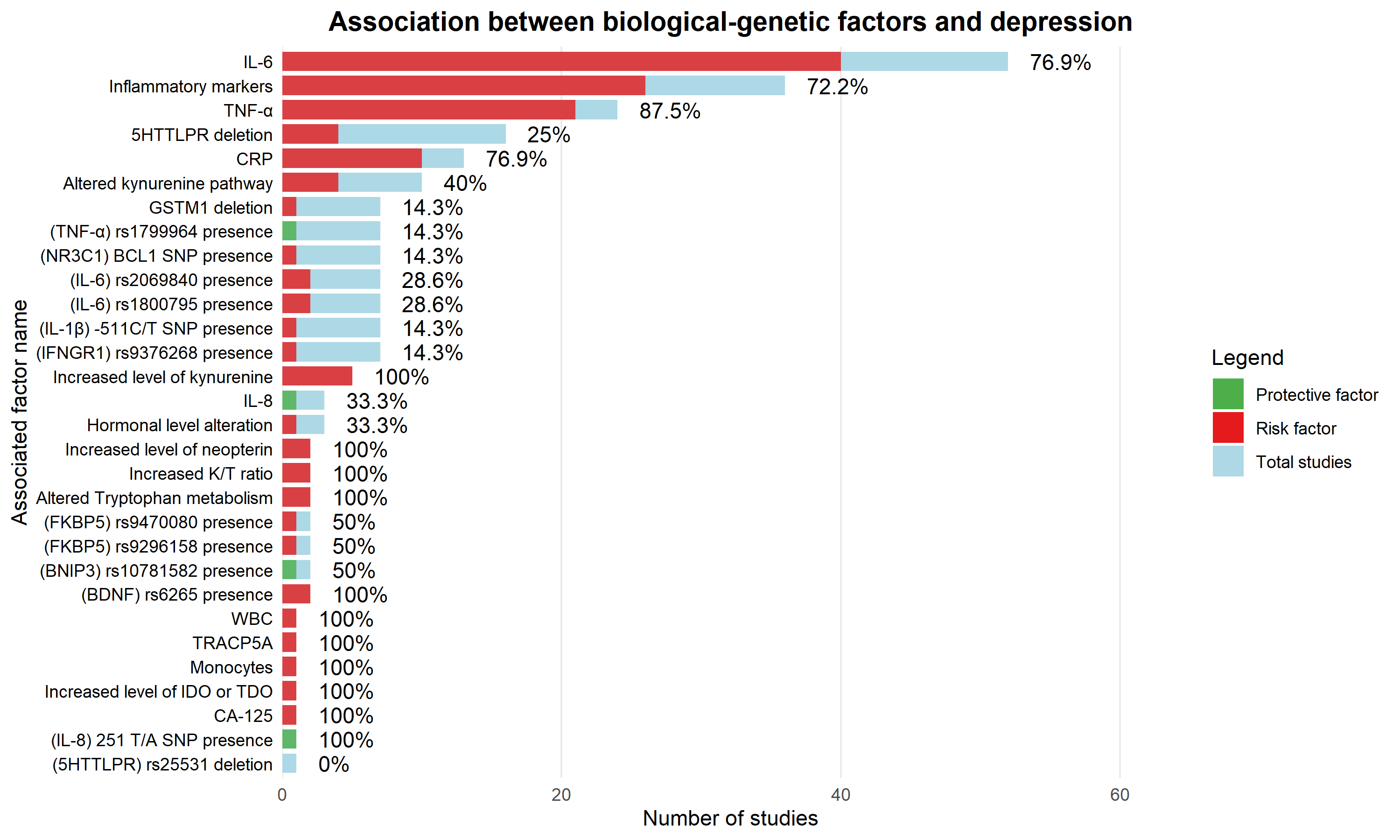


**Table S4. DAFs from Systematic Reviews and Meta-Analyses**

| **Domain** | **Risk or protective** | **Risk factor name** | **Positive associations** | **Total studies** | **Percentage** |
| --- | --- | --- | --- | --- | --- |
| Sociodemographic | Protective | High educational level | 5 | 15 | 33,30% |
| Sociodemographic | Protective | Being employed | 1 | 6 | 16,70% |
| Sociodemographic | Protective | Higher income | 1 | 3 | 33,30% |
| Sociodemographic | Protective | Living with children | 1 | 1 | 100% |
| Sociodemographic | Protective | Being married | 1 | 7 | 14,30% |
| **Sociodemographic** | **Protective** | **High social suport** | **21** | **22** | **95,50%** |
| Sociodemographic | Risk | Older age | 26 | 107 | 24,30% |
| Sociodemographic | Risk | Low educational level | 13 | 40 | 32,50% |
| Sociodemographic | Risk | Being unemployed | 4 | 14 | 28,60% |
| Sociodemographic | Risk | African-American ethnicity | 1 | 11 | 9,10% |
| Sociodemographic | Risk | Asian ethnicity | 1 | 1 | 100% |
| Sociodemographic | Risk | Caucasian ethnicity | 2 | 11 | 18,20% |
| Sociodemographic | Risk | Hispanic ethnicity | 2 | 3 | 66,70% |
| Sociodemographic | Risk | Nuclear family structure | 1 | 1 | 100% |
| Sociodemographic | Risk | Female gender | 31 | 84 | 36,90% |
| Sociodemographic | Risk | Lower income | 18 | 43 | 41,90% |
| Sociodemographic | Risk | Living alone | 15 | 38 | 39,50% |
| **Sociodemographic** | **Risk** | **Being unmarried** | **14** | **14** | **100%** |
| Sociodemographic | Risk | Low social support | 11 | 15 | 73,30% |
| Biological-Genetic | Protective | IL-8 | 1 | 3 | 33,30% |
| **Biological-Genetic** | **Risk** | **TNF-α** | **21** | **24** | **87,50%** |
| **Biological-Genetic** | **Risk** | **CRP** | **10** | **13** | **76,90%** |
| **Biological-Genetic** | **Risk** | **IL-6** | **40** | **52** | **76,90%** |
| Biological-Genetic | Risk | Inflammatory markers | 26 | 36 | 72,20% |
| Biological-Genetic | Risk | CA-125 | 1 | 1 | 100% |
| Biological-Genetic | Risk | TRACP5A | 1 | 1 | 100% |
| Biological-Genetic | Risk | Monocytes | 1 | 1 | 100% |
| Biological-Genetic | Risk | WBC | 1 | 1 | 100% |
| Biological-Genetic | Risk | Hormonal level alteration | 1 | 3 | 33,30% |
| Biological-Genetic | Risk | Increased level of IDO or TDO | 1 | 1 | 100% |
| Biological-Genetic | Risk | Increased K/T ratio | 2 | 2 | 100% |
| **Biological-Genetic** | **Risk** | **Increased level of kynurenine** | **5** | **5** | **100%** |
| Biological-Genetic | Risk | Altered kynurenine pathway | 4 | 10 | 40% |
| Biological-Genetic | Risk | Increased level of neopterin | 2 | 2 | 100% |
| Biological-Genetic | Risk | Altered Tryptophan metabolism | 2 | 2 | 100% |
| Biological-Genetic | Protective | (BNIP3) rs10781582 presence | 1 | 2 | 50% |
| Biological-Genetic | Protective | (IL-8) 251 T/A SNP presence | 1 | 1 | 100% |
| Biological-Genetic | Protective | (TNF-α) rs1799964 presence | 1 | 7 | 14,30% |
| Biological-Genetic | Risk | (IL-6) rs1800795 presence | 2 | 7 | 28,60% |
| Biological-Genetic | Risk | (IL-6) rs2069840 presence | 2 | 7 | 28,60% |
| Biological-Genetic | Risk | (BDNF) rs6265 presence | 2 | 2 | 100% |
| Biological-Genetic | Risk | 5HTTLPR deletion | 4 | 16 | 25% |
| Biological-Genetic | Risk | (FKBP5) rs9296158 presence | 1 | 2 | 50% |
| Biological-Genetic | Risk | (FKBP5) rs9470080 presence | 1 | 2 | 50% |
| Biological-Genetic | Risk | GSTM1 deletion | 1 | 7 | 14,30% |
| Biological-Genetic | Risk | (IFNGR1) rs9376268 presence | 1 | 7 | 14,30% |
| Biological-Genetic | Risk | (IL-1β) -511C/T SNP presence | 1 | 7 | 14,30% |
| Biological-Genetic | Risk | (NR3C1) BCL1 SNP presence | 1 | 7 | 14,30% |
| Biological-Genetic | Risk | (5HTTLPR) rs25531 deletion | 0 | 1 | 0% |
| Somatic | Protective | Frequent sexual activity | 1 | 1 | 100% |
| Somatic | Protective | Physical activity | 1 | 2 | 50% |
| Somatic | Protective | Satisfaction with body image | 1 | 2 | 50% |
| Somatic | Protective | High functional status | 2 | 2 | 100% |
| **Somatic** | **Risk** | **Low functional status** | **18** | **20** | **90%** |
| Somatic | Risk | Negative health behaviors | 6 | 12 | 50% |
| Somatic | Risk | Low Karnofsky performance status | 2 | 4 | 50% |
| **Somatic** | **Risk** | **Physical comorbidities** | **31** | **40** | **77,50%** |
| Somatic | Risk | Cancer-related physical symptoms | 17 | 28 | 60,70% |
| Somatic | Risk | Dry mouth | 1 | 2 | 50% |
| **Somatic** | **Risk** | **Fatigue** | **8** | **9** | **88,90%** |
| Somatic | Risk | Gastrointestinal symptoms | 0 | 2 | 0% |
| Somatic | Risk | Menopausal symptoms | 1 | 1 | 100% |
| Somatic | Risk | Numbness | 0 | 1 | 0% |
| Somatic | Risk | Pain | 18 | 29 | 62,10% |
| Somatic | Risk | Pain duration | 2 | 3 | 66,70% |
| **Somatic** | **Risk** | **Pain intensity** | **4** | **5** | **80%** |
| Somatic | Risk | Restless Leg Syndrome | 2 | 2 | 100% |
| Somatic | Risk | Sense problems | 1 | 2 | 50% |
| Somatic | Risk | Speech problems | 1 | 2 | 50% |
| **Somatic** | **Risk** | **Poor sexual functioning** | **4** | **5** | **80%** |
| Somatic | Risk | Altered masculinity | 2 | 2 | 100% |
| Somatic | Risk | Neck disability | 1 | 1 | 100% |
| Other | Protective | Healthy nutrition | 3 | 3 | 100% |
| **Other** | **Risk** | **Malnutrition** | **8** | **8** | **100%** |
| Other | Risk | COVID-19 pandemic | 0 | 1 | 0% |
| Other | Risk | Family history of cancer | 0 | 1 | 0% |
| Other | Risk | Out of pocket payment | 1 | 10 | 10% |
| Other | Risk | Abortion | 0 | 1 | 0% |
| **Other** | **Risk** | **Sleep issues** | **4** | **5** | **80%** |
| Other | Risk | Alcohol use | 2 | 6 | 33,30% |
| Other | Risk | Smoking | 3 | 8 | 37,50% |
| Cancer-related | Protective | Longer time since surgery | 1 | 1 | 100% |
| Cancer-related | Protective | Late survivorship stage | 1 | 9 | 11,10% |
| Cancer-related | Protective | Longer time since diagnosis | 6 | 18 | 33,30% |
| Cancer-related | Protective | Longer time since chemotherapy | 2 | 14 | 35,70% |
| Cancer-related | Protective | Active surveillance | 6 | 32 | 19% |
| Cancer-related | Protective | Surgery: Lobectomy | 4 | 28 | 14,30% |
| Cancer-related | Protective | Surgery: ''definitive'' treatment | 1 | 1 | 100,00% |
| Cancer-related | Protective | Androgen Receptor Target Therapy (ARAT) (vs Prednisone) | 2 | 2 | 100% |
| Cancer-related | Protective | Androgen Receptor Target Therapy (ARAT) (vs placebo) | 4 | 7 | 57,10% |
| Cancer-related | Protective | Androgen Receptor Target Therapy (ARAT) (vs bicalutamide) | 0 | 2 | 0% |
| Cancer-related | Protective | Abitaterone (ARAT) (vs Enzalutamide, ARAT) | 2 | 3 | 66,70% |
| Cancer-related | Risk | Shorter time since surgery | 1 | 9 | 11,10% |
| **Cancer-related** | **Risk** | **Active surveillance** | **1** | **1** | **100,00%** |
| Cancer-related | Risk | Surgery: Total mastectomy | 18 | 25 | 72,00% |
| Cancer-related | Risk | Surgery: Thyroidectomy/Hemithyroidectomy | 2 | 56 | 3,60% |
| Cancer-related | Risk | Surgery: not specified | 0 | 3 | 0% |
| Cancer-related | Risk | Androgen Deprivation Therapy (ADT) | 25 | 36 | 69,40% |
| Cancer-related | Risk | Taxane therapy | 3 | 8 | 37,50% |
| Cancer-related | Risk | Pharmacological treatment | 1 | 8 | 12,50% |
| Cancer-related | Risk | Adjuvant therapy | 1 | 1 | 100% |
| Cancer-related | Risk | Chemotherapy | 8 | 17 | 47,10% |
| Cancer-related | Risk | Radiotherapy | 12 | 36 | 33,30% |
| Cancer-related | Risk | Use of pain killers | 1 | 2 | 50% |
| Cancer-related | Risk | Being inpatient | 2 | 2 | 100% |
| Cancer-related | Risk | Treatment complications | 6 | 86 | 7% |
| Cancer-related | Risk | High TNM or metastases | 19 | 64 | 29,70% |
| Cancer-related | Risk | Cancer status: active or recurrence | 3 | 11 | 27,30% |
| Cancer-related | Risk | Specified tumor location | 2 | 13 | 15,40% |
| Cancer-related | Risk | Negative cancer perception | 17 | 49 | 34,70% |
| Psychological | Risk | Communication dysfunctions | 1 | 1 | 100% |
| **Psychological** | **Protective** | **Communication: dedicated information time** | **5** | **5** | **100%** |
| Psychological | Risk | Cognitive issues | 3 | 3 | 100% |
| Psychological | Protective | Adaptive coping | 2 | 2 | 100% |
| Psychological | Risk | Maladaptive coping | 7 | 14 | 50% |
| **Psychological** | **Protective** | **Hope level: high** | **14** | **14** | **100%** |
| **Psychological** | **Risk** | **Hope level: low** | **3** | **3** | **100%** |
| **Psychological** | **Risk** | **Life events** | **4** | **5** | **80%** |
| Psychological | Protective | Personality trait: agreeableness | 1 | 1 | 100% |
| Psychological | Protective | Personality trait: high locus of control | 1 | 2 | 50% |
| Psychological | Protective | Personality trait: optimism | 2 | 2 | 100% |
| Psychological | Protective | Personality trait: spirituality | 3 | 3 | 100% |
| Psychological | Protective | Personality trait: self esteem | 2 | 2 | 100% |
| **Psychological** | **Risk** | **Personality trait: introverted** | **9** | **10** | **90%** |
| **Psychological** | **Risk** | **Personality trait: neuroticism** | **5** | **5** | **100%** |
| Psychological | Risk | Personality trait: sense of humor | 1 | 1 | 100% |
| **Psychological** | **Risk** | **History of depression** | **24** | **26** | **92,30%** |
| Psychological | Risk | History of emotional dysfunction | 4 | 4 | 100% |
| Psychological | Risk | History of psychiatric disorder | 13 | 20 | 65% |
| **Psychological** | **Risk** | **Anxiety** | **11** | **11** | **100%** |
| **Psychological** | **Risk** | **Distress** | **6** | **6** | **100%** |
| **Psychological** | **Risk** | **Rumination** | **13** | **15** | **86,70%** |

**Table S5. Prospective DAFs**

| **Domain** | **Risk or protective** | **Risk factor name** | **Positive associations** | **Total studies** | **Percentage** |
| --- | --- | --- | --- | --- | --- |
| Sociodemographic | Protective | High educational level | 1 | 10 | 10,00% |
| Sociodemographic | Protective | Being employed | 1 | 6 | 16,70% |
| Sociodemographic | Protective | Higher income | 1 | 3 | 33,30% |
| Sociodemographic | Protective | Being married | 1 | 7 | 14,30% |
| **Sociodemographic** | **Protective** | **High social support** | **8** | **9** | **88,90%** |
| Sociodemographic | Risk | Older age | 3 | 29 | 10,30% |
| Sociodemographic | Risk | Low educational level | 1 | 1 | 100,00% |
| Sociodemographic | Risk | Nuclear family structure | 1 | 1 | 100% |
| Sociodemographic | Risk | Female gender | 9 | 24 | 37,50% |
| Sociodemographic | Risk | Lower income | 1 | 1 | 100,00% |
| Sociodemographic | Risk | Living alone | 2 | 5 | 40,00% |
| Somatic | Protective | Satisfaction with body image | 1 | 2 | 50% |
| Somatic | Risk | Low Karnofsky performance status | 2 | 4 | 50% |
| Somatic | Risk | Physical comorbidities | 3 | 6 | 50,00% |
| **Somatic** | **Risk** | **Cancer-related physical symptoms** | **5** | **6** | **83,30%** |
| Somatic | Risk | Dry mouth | 1 | 2 | 50% |
| Somatic | Risk | Fatigue | 1 | 1 | 100,00% |
| Somatic | Risk | Gastrointestinal symptoms | 0 | 1 | 0% |
| Somatic | Risk | Pain | 1 | 3 | 33,30% |
| Somatic | Risk | Sense problems | 1 | 2 | 50% |
| Somatic | Risk | Speech problems | 1 | 2 | 50% |
| Somatic | Risk | Poor sexual functioning | 1 | 2 | 50% |
| Somatic | Risk | Neck disability | 1 | 1 | 100% |
| Other | Risk | Malnutrition | 2 | 2 | 100% |
| Other | Risk | COVID-19 pandemic | 0 | 1 | 0% |
| Other | Risk | Abortion | 0 | 1 | 0% |
| Other | Risk | Alcohol use | 1 | 5 | 20,00% |
| Other | Risk | Smoking | 2 | 7 | 28,60% |
| Cancer-related | Protective | Longer time since surgery | 1 | 1 | 100% |
| Cancer-related | Protective | Active surveillance | 6 | 32 | 19% |
| Cancer-related | Protective | Surgery: Lobectomy | 1 | 1 | 100,00% |
| Cancer-related | Risk | Surgery: Thyroidectomy/Hemithyroidectomy | 2 | 26 | 7,70% |
| Cancer-related | Risk | Androgen Deprivation Therapy (ADT) | 10 | 19 | 52,60% |
| Cancer-related | Risk | Taxane therapy | 3 | 8 | 37,50% |
| Cancer-related | Risk | Pharmacological treatment | 1 | 8 | 12,50% |
| Cancer-related | Risk | Chemotherapy | 4 | 11 | 36,40% |
| Cancer-related | Risk | Radiotherapy | 4 | 18 | 22,20% |
| Cancer-related | Risk | Use of pain killers | 1 | 2 | 50% |
| Cancer-related | Risk | Treatment complications | 4 | 27 | 15% |
| Cancer-related | Risk | High TNM or metastases | 8 | 22 | 36,40% |
| Cancer-related | Risk | Specified tumor location | 1 | 8 | 12,50% |
| Cancer-related | Risk | Negative cancer perception | 5 | 9 | 55,60% |
| Psychological | Risk | Communication dysfunctions | 1 | 1 | 100% |
| **Psychological** | **Protective** | **Communication: dedicated information time** | **5** | **5** | **100%** |
| Psychological | Risk | Maladaptive coping | 2 | 8 | 25% |
| Psychological | Risk | Life events | 2 | 3 | 67% |
| Psychological | Protective | Personality trait: high locus of control | 1 | 2 | 50% |
| Psychological | Protective | Personality trait: spirituality | 3 | 3 | 100% |
| Psychological | Protective | Personality trait: self esteem | 1 | 1 | 100% |
| Psychological | Risk | Personality trait: sense of humor | 1 | 1 | 100% |
| **Psychological** | **Risk** | **History of depression** | **8** | **10** | **80,00%** |
| Psychological | Risk | Anxiety | 3 | 3 | 100% |
| Psychological | Risk | Distress | 1 | 1 | 100% |
| Psychological | Risk | Rumination | 3 | 3 | 100,00% |
